# Dense attention network identifies EEG abnormalities during working memory performance of patients with schizophrenia

**DOI:** 10.3389/fpsyt.2023.1205119

**Published:** 2023-09-25

**Authors:** Ruben Perellón-Alfonso, Aleš Oblak, Matija Kuclar, Blaž Škrlj, Indre Pileckyte, Borut Škodlar, Peter Pregelj, Kilian Abellaneda-Pérez, David Bartrés-Faz, Grega Repovš, Jurij Bon

**Affiliations:** ^1^Faculty of Medicine and Health Sciences, and Institute of Neurosciences, University of Barcelona, Barcelona, Spain; ^2^Institute of Biomedical Research August Pi i Sunyer (IDIBAPS), Barcelona, Spain; ^3^University Psychiatric Clinic Ljubljana, Ljubljana, Slovenia; ^4^Department of Psychiatry, Faculty of Medicine, University of Ljubljana, Ljubljana, Slovenia; ^5^Jožef Stefan Institute, Ljubljana, Slovenia; ^6^Center for Brain and Cognition, Pompeu Fabra University, Barcelona, Spain; ^7^Institut Guttmann, Institut Universitari de Neurorehabilitació Adscrit a la UAB, Barcelona, Spain; ^8^Department of Psychology, Faculty of Arts, University of Ljubljana, Ljubljana, Slovenia

**Keywords:** schizophrenia, working memory (WM), contralateral delay activity (CDA), electroencephalography (EEG), dense attention network (DAN)

## Abstract

**Introduction:**

Patients with schizophrenia typically exhibit deficits in working memory (WM) associated with abnormalities in brain activity. Alterations in the encoding, maintenance and retrieval phases of sequential WM tasks are well established. However, due to the heterogeneity of symptoms and complexity of its neurophysiological underpinnings, differential diagnosis remains a challenge. We conducted an electroencephalographic (EEG) study during a visual WM task in fifteen schizophrenia patients and fifteen healthy controls. We hypothesized that EEG abnormalities during the task could be identified, and patients successfully classified by an interpretable machine learning algorithm.

**Methods:**

We tested a custom dense attention network (DAN) machine learning model to discriminate patients from control subjects and compared its performance with simpler and more commonly used machine learning models. Additionally, we analyzed behavioral performance, event-related EEG potentials, and time-frequency representations of the evoked responses to further characterize abnormalities in patients during WM.

**Results:**

The DAN model was significantly accurate in discriminating patients from healthy controls, *ACC* = 0.69, SD = 0.05. There were no significant differences between groups, conditions, or their interaction in behavioral performance or event-related potentials. However, patients showed significantly lower alpha suppression in the task preparation, memory encoding, maintenance, and retrieval phases *F*(1,28) = 5.93, *p* = 0.022, η^2^ = 0.149. Further analysis revealed that the two highest peaks in the attention value vector of the DAN model overlapped in time with the preparation and memory retrieval phases, as well as with two of the four significant time-frequency ROIs.

**Discussion:**

These results highlight the potential utility of interpretable machine learning algorithms as an aid in diagnosis of schizophrenia and other psychiatric disorders presenting oscillatory abnormalities.

## 1. Introduction

Schizophrenia is a severe neuropsychiatric disorder with a global prevalence of 0.28% and a significant socioeconomic burden (1). The symptoms of schizophrenia can be divided into positive (i.e., hallucinations, delusions and disorganized thinking) and negative [i.e., decreased emotional expression, social withdrawal, and cognitive impairments of memory and executive functions; (2, 3)]. Schizophrenia is thought to be a neurodevelopmental disorder caused by interaction of genetic and early environmental risk factors (4–6), resulting in impaired large-scale connectivity (7, 8) and aberrant brain activity (9, 10). Pathophysiological changes include altered dopamine and glutamate neurotransmission, which is thought to be related to a disruption in the balance of excitation and inhibition in cortical microcircuits, contributing to altered synchronization of neuronal oscillations (11).

Impairment of working memory (WM) is a core cognitive deficit in schizophrenia that significantly correlates with functional capacity and outcome (12), and has been proposed as a warning sign of conversion to psychosis (13). WM is often defined as a system with limited capacity for the temporary storage and manipulation of representations of information necessary to guide behavior in complex goal-directed tasks such as comprehension, learning, and reasoning (14), and it overlaps with other cognitive domains such as attention and executive function (15). In schizophrenia, deficits can be observed in all WM subprocesses and stimulus types (16), and are associated with impairments in proactive cognitive control [i.e., the ability to actively represent goal information in working memory to guide behavior (16)] or attention hyperfocus [i.e., an abnormally narrow and intense focusing of processing resources; (17)]. Deficits have also been detected, in high-functioning patients with preserved WM performance, in the form of increased reaction time variability (18), which has been interpreted as impaired information processing. The visual modality of WM is particularly relevant in schizophrenia, as it strongly correlates with measures of higher cognitive functions and, according to some estimates, may account for up to 40% of the cognitive deficit in patients with schizophrenia (19).

Working memory tasks can be constructed to engage different WM subprocesses either simultaneously [e.g., N-back tasks; (20, 21)] or sequentially [e.g., verbal span tasks, visuospatial change detection tasks; (22, 23)]. Sequential tasks are particularly useful to probe behavioral performance and brain activity during separate time periods of the WM task corresponding to task preparation, encoding, maintenance, and retrieval of information, all of which have been shown to be affected in schizophrenia (24–26).

Electroencephalographic (EEG) studies of event-related potentials (ERPs) elicited during working memory tasks, have shown abnormalities in electrical activity during early evoked responses and late, cognition-related components of schizophrenia patients (27). In visual WM, a lateralized change detection task (23) elicits a corresponding ERP component, the contralateral delay activity (CDA), which has been shown to be closely related to WM capacity and is modulated by load (28). CDA studies in schizophrenia have shown that visual WM capacity is lower, relative to healthy controls, and that patients also show specific impairments in attention control during the task (29). In addition to ERP abnormalities, studies also found changes on synchronized neuronal oscillations in several frequency bands. Specifically, gamma (>30 Hz), which is involved in sensory processing (30) and maintenance of WM information (31), shows lack of synchronization in schizophrenia patients during WM tasks [e.g., (32)]. Theta (4–7 Hz), which supports long range connectivity and coordination of WM items (33), has been reported to be abnormally high during resting state (34) and decoupled from gamma during WM performance (35). Finally, alpha (8–12 Hz) desynchronization (also known as alpha suppression), which reflects the active inhibition of task-irrelevant information (36, 37), has been shown to be impaired in schizophrenia patients and individuals at risk of psychosis during working memory and oddball tasks (24, 38–41).

While these studies have significantly advanced our understanding of the neurophysiological basis of schizophrenia, they typically rely on univariate statistical methods that, while suitable for group-level comparisons, are insufficient for the purposes of individual diagnosis within the framework of precision psychiatry (42, 43). Moreover, these studies highlight the fact that schizophrenia exhibits heterogenic symptoms and intricate neurophysiological foundations that cannot be attributed to a single brain area or neural process and that might be shared across psychiatric disorders (44). This complexity makes precise differential diagnosis and neurophysiological characterization of individual patients challenging. To confront these challenges, the field of psychiatry has increasingly turned to machine learning, a class of artificial intelligence approaches where algorithms are designed to make successful predictions without explicit programming (45). A growing number of studies have used EEG data to successfully classify patients and controls with high accuracy (46–52). These results have the potential to yield clinically translatable improvements in diagnosis. However, the best performance is often achieved by deep convolutional neural network models, which are said to be “black boxes,” since there is no straightforward solution to disentangle how the algorithm transforms the input data to model a particular output (53). This characteristic limits their utility for investigating the neurophysiological substrate of schizophrenia and identifying suitable biomarkers for early detection, consequently, more transparent and interpretable deep learning models are needed to fill this gap (54).

A promising alternative is the dense attention network (DAN), a type of deep learning model based on the attention model (55), a simple mechanism that scatters input signals and highlights only the parts of the feature space that are relevant to the task at hand. Crucially, the attention layers can output a probability distribution over the input space, thus providing an insight into the inner workings of the neural network, in the form of a one-to one mapping of the relative contribution of each feature in the input space (56, 57).

Here, we took a data-driven machine learning approach to determine the distribution of EEG signatures specific to schizophrenia patients over the time course of a visuospatial change detection task. Based on previous encouraging reports (46–52), we hypothesized that machine learning could be used to successfully classify patients from controls based on EEG alone. We chose an interpretable subtype of machine learning based on the attention model (55), with the hypothesis that specific temporal signatures would be most discriminative of patients and controls. We hypothesized that differences between schizophrenia patients and control subjects would also be evident using univariate statistical methods, particularly on oscillatory activity related to attention control (i.e., in the alpha frequency band), which has been consistently reported to be impaired in patients with schizophrenia, and would be most prominent during the task preparation, encoding, maintenance or memory retrieval phases of the task time-course (24, 39, 41). Finally, we expected this significantly different task segments to overlap with the features found to be most discriminative by the DAN model. This correspondence is of crucial importance if machine learning is to become not just a diagnostic aid, but also a tool capable of proving the neurophysiological substrate of schizophrenia (54).

## 2. Materials and methods

### 2.1. Study participants

Fifteen patients with a mean age of 28.1 years, *SD* = 3.9, and an average of 13.4 years of education, *SD* = 1.1, were recruited from the Department for Psychotherapy of Psychotic Disorders at the University Psychiatric Clinic Ljubljana (see Supplementary Table 1 for descriptive statistics on demographics). All participants included in the study were male, due to a lack of a representative number of female participants available at the time of recruitment. All patients had a diagnosis of schizophrenia (12 subjects) or schizoaffective disorder (3 subjects). The diagnoses were confirmed according to the DSM-IV criteria by experienced clinicians (BŠ and JB) involved in the study. At the time of the experiment, all patients were taking second generation antipsychotic medication and were in stable symptomatic remission, with an average PANSS score (58) of 77.1 (*SD* = 15.3), and were cleared for inclusion in psychodynamic group psychotherapy. The patients’ mean duration of illness was 6.1 years (*SD* = 3.3), and the mean number of hospitalizations was 2.9 (*SD* = 2.1). For further clinical details, see Supplementary Table 1.

Additionally, we recruited a control group of 15 male participants of comparable age, *M* = 26.8 years, *SD* = 5.5, and years of education, *M* = 14.4, *SD* = 1.2. The study was approved by the Medical Ethics Committee of the Republic of Slovenia and all participants signed an informed consent form according to the Declaration of Helsinki.

### 2.2. Visual working memory task and EEG recording

**FIGURE 1:**
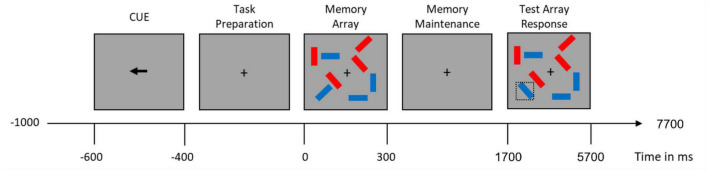
Visual working memory trial time-course. Doted square containing the blue item on the lower left part of the test array illustrates an item that changed from the memory array.

A lateralized change detection task with distractors (23, 59) was implemented using PsychoPy (60). First, participants were shown an arrow cue for 200 ms, the direction of which indicated to which half of the visual field they should direct their attention. This was followed first by a fixation cross shown for 400 ms and then by a memory array shown for 300 ms. The memory array consisted of 2 or 4 rectangles (in each half of the visual field). The rectangles were colored either blue, or blue and red (in the distractor condition), and were shown in one of 4 possible orientations (0°, 45°, 90°, or 135°). Participants were asked to remember the orientations of the blue rectangles shown on the cued side of the visual field. The presentation of the memory array was followed by a delay of 1400 ms before the presentation of a test array, that remained for 4 s and was then followed by 2 s without any stimulus. The test array was either identical to the memory array or with only one of the randomly selected (blue) rectangles on the cued side changing its orientation in half of the trials. The participants’ task was to indicate whether any of the target items had changed by pressing the corresponding button on a response box (Figure 1).

There were three task conditions, differing in the number of target items and the presence of a distractor:

1. A condition with two blue rectangles shown on each side (low memory load; condition 2),
2. A condition with four blue rectangles shown on each side (high memory load: condition 4), and
3. A condition with two blue and two red rectangles shown on each side (distractor condition; condition 2+2).

In the 2+2 condition, participants had to successfully inhibit the two red distractor rectangles presented together with the two blue memory rectangles. The trials belonging to the different conditions were interleaved within a block. Participants were familiarized with the task during the practice trials, and the experimenter ensured that they all performed with at least 70% accuracy.

Participants performed 200 trials for each of the three conditions in an electrically shielded and soundproofed room while seated in a comfortable chair in front of a cathode ray monitor. Throughout the task, the EEG signal was recorded using four BrainAmp amplifiers connected to a 128-channel actiCAP system with active electrodes in a standard montage (Brain Products GmbH, Munich, Germany). The EEG was recorded with a 2000 Hz low-pass filter and digitized at a sampling rate of 500 Hz.

### 2.3. Working memory task performance metrics

To compare behavioral task performance between groups we computed memory capacity index K (61) and intra-individual reaction time variability (see Supplementary Table 2 for detailed descriptive statistics of task performance).

#### 2.3.1. Working memory capacity

WM capacity index K was calculated for each subject and condition using the Pashler variant of the formula appropriate for a whole-display variant of a change detection task (61):


$$K=N\,\left(\frac{H\,R-F\,A\,R}{1-F\,A\,R}\right)$$

Where *HR* is the hit rate, *FAR* is the false alarm rate, and *N* is the number of to-be-remembered items.

#### 2.3.2. Intra-individual reaction time variability

Rentrop and colleagues (18) reported schizophrenia patients with relatively well-preserved WM performance still showed higher intraindividual variability in reaction times. Therefore, we compared the coefficient of variation of reaction times between the two groups, which was defined as the ratio of the standard deviation to the mean of the reaction times.

### 2.4. EEG preprocessing

Electroencephalographic data were preprocessed using EEGLAB functions (62) and custom-made MATLAB (The MathWorks Inc., Massachusetts, USA) scripts. Data were first filtered with a high-pass filter with a 0.5 Hz frequency cutoff, then the line frequency noise was removed from the signal using the CleanLine algorithm (63). Visual inspection was aided by statistical thresholding based on variance and Kurtosis to identify bad channels, *M* = 15.3, *SD* = 4.6. Next data were referenced to the average of the mastoid channels (i.e., TP9 and TP10) and segmented into epochs around the onset of the memory array (−1,000 ms to 4,500 ms). At this point, epoched data were visually inspected, and epochs that contained obvious artifacts (e.g., high-frequency or muscular artifacts) were removed. Because lateral eye movements would impact the magnitude of the CDA, electrooculogram channels were visually inspected for the time period from the presentation of arrow cue to the presentation of memory array, and all epochs with eye blinks or horizontal eye movements in this period were also discarded, bringing the total average of epochs removed to 47.6.3, *SD* = 29.5. Next, the AMICA algorithm (64) was used to identify and then remove any remaining artifactual independent components *M* = 6.4, *SD* = 2.4. Last, the channels previously removed from the data were spline interpolated based on the signal from the neighboring electrodes.

### 2.5. Machine learning methods and empirical evaluation

The aim of this analysis was to investigate the potential of machine learning methods to discriminate patients from controls. Given the heterogeneous nature of schizophrenia, our aim was to produce a model capable of discriminating between patients and controls without relying on any specific clinical data, leveraging only EEG data that has been preprocessed using relatively simple and well-established procedures. The dense attention network model was deliberately chosen because it retains a sufficient level of interpretability to explain which events were most important in distinguishing patients from controls over the time course of the experiment (57) The dimensionality reduction of the data, the construction of the DAN architecture, and its evaluation, were performed using in house methods (a detailed description can be found in Supplementary material; scripts and data used to design, train, and evaluate the different machine learning models^1^). Briefly, the dimensionality of the preprocessed data was reduced from 4d to 1d by incremental stepwise averaging of three of the four original dimensions (i.e., 3 conditions left and 3 right, 128 channels, 2,750 time-points and 153 trials on average):


$$S_{\gamma }=\frac{1}{R\,N\,Z}\,\sum_{\mu =1}^{R}\left(\sum_{\nu =1}^{N}\left(\sum_{\sigma =1}^{Z}M_{\mu \,\nu \,\gamma \,\sigma }\right)\right)$$

Where μ, ν, γ, and σ stand for condition, channel, time, and trial, respectively. In this way, a 1d array was created for each subject while preserving the temporal characteristics of the data. This simplified dimensionality of the data allowed us to train a dense attention network model (DAN). The final input dataset used in the model was numeric and consisted of 30 instances (i.e., number of subjects), each described by 2,750 features (i.e., corresponding to the preserved time dimension after the incremental stepwise averaging of the original 4d EEG data set).

Empirical evaluation of model performance consisted of leave-one-out cross-validation repeated ten times for each model. For the DAN model there were 160 possible configurations evaluated. We used the Adam optimization algorithm (65) and we considered the following parameters: dropout rate (0.01, 0.05, 0.2, and 0.5) hidden layer size (16, 32, 64, and 128), number of epochs (2, 4, 8, 16, and 32) and learning rate (0.001 and 0.0001).

The performance of the model was compared with the performance of other simpler architectures (i.e., linear regression, radio frequency machine learning, support vector machine, radial basis function and k-nearest neighbor), and common deep learning models (convolutional neural network and feed forward neural network).

We report the average and standard deviation of the resulting accuracy, precision and recall from these iterations. We also report the *F*_1_ scores, computed as follows:


$$F_{1}=2\,\frac{p\,r\,e\,c\,i\,s\,i\,o\,n\cdot r\,e\,c\,a\,l\,l}{p\,r\,e\,c\,i\,s\,i\,o\,n+r\,e\,c\,a\,l\,l}$$

All models were implemented using the PyTorch deep learning library (66) and evaluated on a Tesla graphics card accelerator (Nvidia Corp. Santa Clara, USA).

Throughout training of the DAN model, a bijection is maintained with the input space (i.e., the attention layer corresponds to the input space in a one-to-one relationship). Therefore, we were able to use the attention layer’s output directly as a probability distribution over the input space. This attention value vector quantifies the contribution of each feature (EEG time-point in the WM task time-course) in the distinction between patients and controls.

### 2.6. Event related potential analysis

**FIGURE 2:**
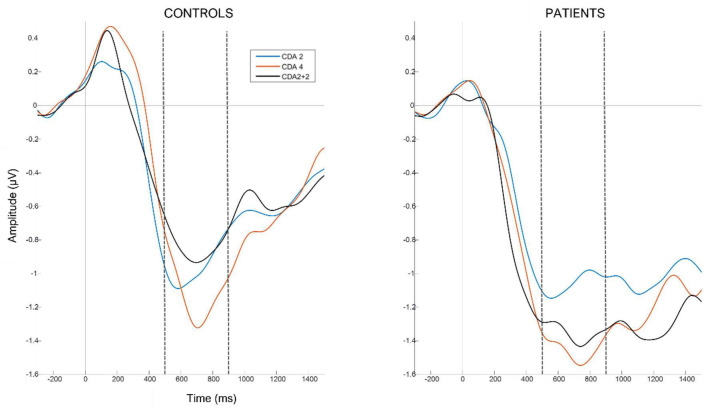
Grand average contralateral delayed activity waveforms per each group (i.e., patients **left panel** and controls **right panel**) and condition (2 items in blue, 4 items in red and 2+2 items in black), averaged over parieto-occipital electrodes. Vertical gray lines at time 0 correspond to the presentation of the memory array. Horizontal gray lines correspond to 0 amplitude. Horizontal doted black lines mark the time segment (500–900 ms after memory array onset) from which we extracted the average CDA amplitude for each subject.

In order to capture the electrophysiological correlate of WM capacity, we computed the contralateral delayed activity (CDA) as the difference between the contralateral and ipsilateral (relative to the cued side for the memory array) ERP waveforms using an established procedure (23, 28). For each subject, the mean amplitude of the resulting CDA difference curves was measured for the average of all parieto-occipital electrodes and the time segment from 500 ms to 900 ms after the presentation of the memory array (Figure 2). The resulting mean CDA amplitude data were used for further statistical analysis.

### 2.7. Time-frequency analysis

To compare the oscillatory dynamics between patients and controls, throughout the trial time course, we performed a time-frequency analysis of total power (i.e., comprising induced and evoked power) for the epoched data. Data was decomposed into the time-frequency domain by convolving a set of complex Morlet wavelets from 1 Hz to 60 Hz, in steps of 1 Hz, with a logarithmically spaced wavelet width of 4–10 cycles. The resulting time-frequency maps were normalized as the decibel (db) change from baseline (i.e., −850 to −650 ms from the memory array presentation). Time-frequency regions of interest (ROIs) were then determined based on the main WM task phases and frequency bands. Specifically, the time segments of interest (i.e., ROIs x-axis) were, *preparation* for the task (−400 to 0 ms), *encoding* (0–300 ms), *maintenance* (300–1,700 ms) and *retrieval* of the memory array (1,700–3,060 ms). The end of the time window for the memory retrieval phase was chosen based on the average reaction time plus one standard deviation in the slower group (i.e., patients; Supplementary Table 2). We included four frequency bands of interest (i.e., ROIs y-axis), theta (4–7 Hz), alpha (8–12 Hz), beta (13–29 Hz) and gamma (30–60 Hz) to explore possible group differences across the frequency spectrum. For further statistical analysis, the average of all time-frequency data points within each ROI for each subject and condition was used.

### 2.8. Statistical analysis

For behavioral analysis a mixed design ANOVA was used for memory capacity K and another for reaction-time variability. For ERP analysis one mixed design ANOVA was used. For time-frequency total power four mixed design ANOVAs were used, one for each frequency band. All statistical tests were implemented in R (67). Greenhouse-Geisser correction was used in case of sphericity violations. All *post-hoc* comparisons were performed with paired or Welch’s *t*-tests with Bonferroni corrections.

## 3. Results

### 3.1. Memory capacity K

To test for differences in memory capacity between groups and conditions we used a mixed design ANOVA with a within-subject factor *condition* (condition 2, condition 2+2 and condition 4) and a between-subject factor *group* (patient vs. control). The test revealed no significant main effect of *group*, *F*(1,28) = 2.21, *p* = 0.149, η^2^ = 0.043, but there was a significant effect of *condition*, *F*(2,56) = 33.74, *p* < 0.001, η^2^ = 0.344. *Post-hoc* analysis with a pairwise *t*-test revealed that in condition 4, *M* = 2.57, *SD* = 0.86, memory capacity K was significantly higher than in conditions 2, *M* = 1.81, *SD* = 0.14, *p* < 0.001, *d* = 1.236 and 2+2, *M* = 1.76, *SD* = 0.32 *p* < 0.001, *d* = 1.245. There was no interaction between *group* and *condition*, *F*(2,56) = 1.13, *p* = 0.329, η^2^ = 0.017.

### 3.2. Reaction time’s coefficient of variation

A mixed design ANOVA with a within-subject factor of *condition* (condition 2, condition 2+2 and condition 4) and a between-subject factor of *group* (patient vs. control) was used to test for differences in the coefficient of variation. The test revealed no significant main effect *condition*, *F*(2,56) = 1.08, *p* = 0.345, η^2^ = 0.007, *group, F*(1,28) = 2.49, *p* = 0.125, η^2^ = 0.068, or their interaction *F*(2,56) = 1.14, *p* = 0.326, η^2^ = 0.007.

### 3.3. Contralateral delay activity’s mean amplitude

To examine the differences in mean CDA amplitude between the two groups and the three experimental conditions, we used a mixed design ANOVA with a within-subject factor *condition* (condition 2, condition 2+2, and condition 4) and a between-subject factor *of group* (patient vs. control). This analysis revealed no significant main effect of *group F*(1,28) = 0.22, *p* = 0.642, η^2^ = 0.004, *condition, F*(2,56) = 3.12, *p* = 0.065, η^2^ = 0.048, or their interaction *F*(2,56) = 0.41, *p* = 0.617, η^2^ = 0.007.

### 3.4. Time-frequency power representation

To investigate possible differences between patients and controls in overall power at four frequency bands, we used a mixed design ANOVA for each frequency band with a within-subject factor *condition* (condition 2, condition 2+2, and condition 4), a within-subject factor WM task *phase* (preparation, encoding, maintenance, retrieval), and a between-subject factor *group* (patient vs. control). This analysis revealed a significant *group* effect, *F*(1,28) = 5.93, *p* = 0.022, η^2^ = 0.149, and interaction between *group*, *condition* and task *phase*, *F*(6,168) = 2.20, *p* = 0.046, η^2^ = 0.002, only for the alpha frequency band (for complete results in the theta, beta, and gamma bands see Supplementary Section 5 and Supplementary Figure 3). *Post-hoc* analysis with Welch’s *t*-test revealed that patients had overall higher alpha power (i.e., less alpha suppression), *M* = −1.04, *SD* = 0.89, than controls, *M* = −2.33, *SD* = 1.84, *t*(20.24) = 2.43, *p* = 0.024, *d* = 0.889.

**FIGURE 3:**
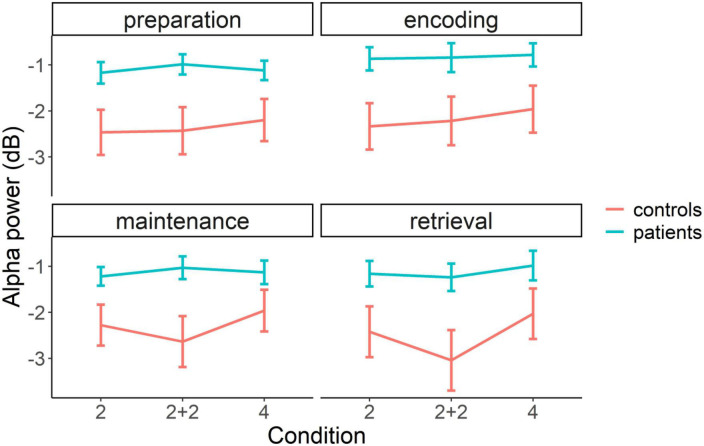
Average alpha power baseline normalized in patient (blue line) and control (red line) groups in three different conditions (2, 2+2, and 4) and four WM task phases (preparation, encoding, maintenance, and retrieval). The error bars represent standard error of the mean.

To unravel the triple interaction, we ran an additional mixed design ANOVA for each of the four WM task phases, with a within-subject factor *condition* (condition 2, condition 2+2, and condition 4) and a between-subject factor *group* (patients vs. controls). We found that groups differed in each of the 4 WM task phases (Figure 3). During task preparation, *F*(1,28) = 6.16, *p* = 0.019, η^2^ = 0.167, patients, *M* = −1.09, *SD* = 0.81, had higher alpha power than controls, *M* = −2.36, *SD* = 1.81, *t*(19.41) = 2.48, *p* = 0.022, *d* = 0.906. During memory encoding, *F*(1,28) = 5.73, *p* = 0.024, η^2^ = 0.159, patients, *M* = −0.83, *SD* = 1.00, had higher alpha power than controls, *M* = −2.17, *SD* = 1.93, *t*(21.04) = 2.39, *p* = 0.026, *d* = 0.874. During memory maintenance, we found a significant interaction between group and condition, *F*(2,56) = 3.28, *p* = 0.045, η^2^ = 0.013. A follow-up analysis revealed that the interaction was driven by the decrease in alpha power from condition 2+2, *M* = −1.03, *SD* = 0.96, to condition 4, *M* = −1.13, *SD* = 0.99, in patients, and increase in alpha power from condition 2+2, *M* = −2.63, *SD* = 2.14, to condition 4, *M* = −1.96, *SD* = 1.75, in controls. Finally, during memory retrieval, *F*(1,28) = 4.73, *p* = 0.038, η^2^ = 0.133, patients, *M* = −1.13, *SD* = 1.13, had higher alpha power than controls, *M* = −2.50, *SD* = 2.16, *t*(21.12) = 2.17, *p* = 0.041, *d* = 0.794.

**FIGURE 4:**
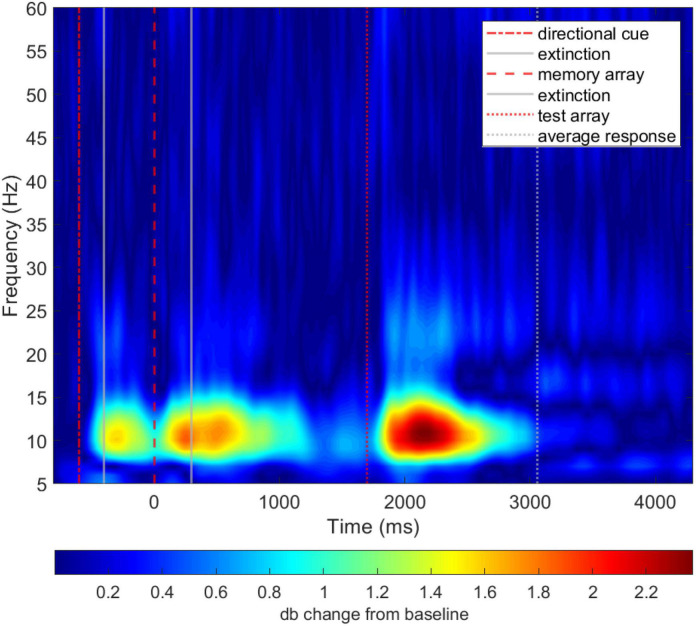
Unthresholded average time-frequency representation of total power difference of patients and controls (i.e., patients–controls).

To summarize, there was a significant difference between patients and controls in the alpha frequency band, but not in other frequency bands. This difference was observed in all four task phases. In support to these univariate results, the difference between patients and controls can also be visually evaluated in time-frequency difference maps, where it can be seen that the differences in alpha band peak after the presentation of the main task stimuli, namely, directional cue, memory array and test array (Figure 4).

### 3.5. Machine learning performance and interpretation

The results from the empirical evaluation show that the DAN model consistently demonstrated accuracy in discriminating patients from controls significantly above chance, *ACC* = 0.69, *SD* = 0.05, *F*_1_ = 0.71, SD = 0.07, *Recall* = 0.77, SD = 0.11, *PRC* = 0.67, SD = 0.03 (Table 1). This model outperformed simpler architectures, such as the support vector machine, while performing similarly to other common deep neural network models, such as the convolutional neural network. Full results for all models tested under different subsets of the feature space (i.e., task conditions) can be found in Supplementary Figure 1 and Supplementary Table 3.

**TABLE 1:** Summary of the empirical evaluation average results for the different machine learning architectures used.

Model	Accuracy	F1	Recall	Precision
DAN	0.69 ± (0.05)	0.71 ± (0.07)	0.77 ± (0.11)	0.67 ± (0.03)
FFNN	0.71 ± (0.03)	0.72 ± (0.03)	0.73 ± (0.05)	0.70 ± (0.02)
CNN	0.69 ± (0.05)	0.72 ± (0.05)	0.81 ± (0.05)	0.66 ± (0.04)
LR	0.65 ± (0.0)	0.67 ± (0.0)	0.69 ± (0.0)	0.64 ± (0.0)
SVM linear	0.62 ± (0.0)	0.62 ± (0.0)	0.62 ± (0.0)	0.62 ± (0.0)
SVM poly	0.62 ± (0.0)	0.58 ± (0.0)	0.54 ± (0.0)	0.64 ± (0.0)
KNN	0.69 ± (0.0)	0.71 ± (0.0)	0.77 ± (0.0)	0.67 ± (0.0)
RF	0.56 ± (0.08)	0.58 ± (0.09)	0.62 ± (0.11)	0.55 ± (0.07)
SVM rbf	0.58 ± (0.0)	0.62 ± (0.0)	0.69 ± (0.0)	0.56 ± (0.0)

Standard deviations in parenthesis. CNN, convolutional neural network; DAN, dense attention network; FFNN, feed forward neural network; KNN, K-nearest neighbor; LR, linear regression; RF, radio frequency machine learning; SVM, support vector machine; rbf, radial basis function.

**FIGURE 5:**
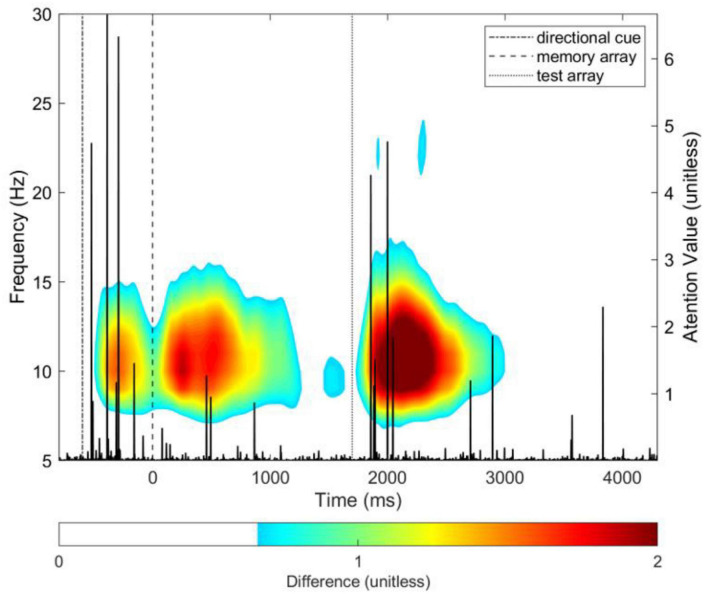
DAN attention value vector (in black; y-axis scale on the right side of plot) overlayed on the group difference time-frequency total power time-frequency map for the average of all parieto-occipital electrodes. The difference was computed by subtracting the grand average map of total power of controls from patients (i.e., patients–controls). The colormap is thresholded, for visualization purposes only, to show no color for values lower than 30% of the maximum difference value of 2. See Figure 4 for unthresholded map.

Projecting the DAN attention value over the WM task timeline and onto the time-frequency map of group differences shows that the time points most relevant to the model’s discrimination between groups (i.e., attention value) overlap with time-frequency ROIs that were found to be significantly different between groups (Figure 5), and correspond to the task preparation and memory retrieval phases of the WM task.

## 4. Discussion

In this study, we investigated the WM performance and associated EEG signatures of schizophrenia patients and compared them to healthy controls. The results show that in our sample neither WM performance, measured by memory index K and reaction time variability, nor CDA amplitude showed a significant difference between patients and controls. However, statistical analysis in the time-frequency domain revealed, a significant group effect in all time segments of interest (task preparation, memory encoding, maintenance and retrieval) in the alpha-band range (8–12 Hz). We demonstrated that a simple dimensionality reduction procedure consisting of incremental stepwise averaging, that preserves the temporal characteristics of the EEG signal, can be used as input to train a DAN machine learning model capable of successfully discriminating patients from control subjects based on the EEG signal after standard preprocessing alone, with accuracy significantly above chance (*ACC* = 0.69). We then compared the model’s performance with simpler machine learning architectures, as well as more common deep neural network models, showing similar performance. Finally, direct mapping of the attention value vector with the WM task trial time course, revealed that the most discriminative time points for the classification overlapped with the task preparation and memory retrieval phases, as well as with the identified time-frequency regions of interest that show significant group differences in alpha suppression, with patients showing less suppression than controls at these ROIs.

### 4.1. Normal WM performance and contralateral delay activity in schizophrenia patients

In our study, behavioral and CDA results did not differ significantly between patients and controls. This is in contrast with previous studies that generally find working memory performance deficits in schizophrenia (68, 69). Recent studies have found associations between poor performance and deficits in consolidation or early maintenance of stimuli (24), deficits in attention and executive control (70) or less efficient allocation of memory resources (71). Previous studies also report CDA amplitude differences between patients and controls, with amplitude being larger than that of control subjects at low memory load but smaller at high memory load (29), even when their maximum visual WM capacity is equal to that of control subjects. This pattern of impairment may support the theory of inefficient attention hyperfocus on a small number of items, especially when they are salient (17).

Normal behavioral and CDA results in our sample suggest that patients performed well on this particular visual WM task. These results are consistent with previous research showing no differences between high-functioning individuals with schizophrenia and healthy controls in task performance (18, 72) and working memory related ERPs (73, 74). Thus, given the preserved working memory performance and lack of significant CDA abnormalities, our findings may be more representative of high-functioning patients. In this context, it is worth noting that, at the time of recruitment and throughout the data gathering phase, our patients were asymptomatic and engaged in psychodynamic group psychotherapy. While this criterion alone need not imply high-functioning status, given the known associations between engagement in psychodynamic psychotherapy and functional outcome (75), it is reasonable to suspect that our patients might potentially be close to high-functioning.

### 4.2. Schizophrenia patients exhibit decreased suppression of alpha spectral power during visual WM task

Our analysis revealed significantly lower suppression of non-lateralized parietal alpha spectral power during the task preparation, memory encoding, maintenance, and retrieval phases of the visual WM change detection task. Given the recognized role of oscillations in the alpha frequency band in long-range synchronization (38), top-down control (76, 77), attention (78) and cortical inhibition (79, 80), our time-frequency results may reflect a deficit that makes it difficult for patients to inhibit task-irrelevant brain regions and processes and to maintain efficient attention control, regardless of the experimental condition. These results are consistent with existing literature reporting alpha suppression abnormalities in schizophrenia during working memory tasks (24, 38–41). In the presence of these potential inhibitory and attention deficits, patients might have been able to maintain behavioral performance through various compensatory strategies, such as greater attention effort (17) reflected by less alpha suppression.

### 4.3. Deep attention networks can discriminate high performing individuals with schizophrenia from healthy controls in EEG data after dimensionality reduction

The dense attention network model implemented in this study was able to classify patients and controls with an accuracy significantly above chance, *ACC* = 0.69 outperforming simpler machine learning architectures, while achieving similar performance to more commonly used deep network models. Moreover, our attention model revealed the relative importance of each feature in the input space for the successful classification of patients and controls. This was possible owing to our proposed data aggregation technique (i.e., incremental stepwise averaging), that allowed us to reduce each patient’s preprocessed EEG data to a one-dimensional vector, while preserving the temporal characteristics of the signal.

The results show that the time points that were most discriminative for the machine learning algorithm, overlapped with both the preparatory and memory retrieval phases during the task, as well as with ROIs selected from the time-frequency maps. Furthermore, the two highest peaks in The DAN attention value vector were found to overlap with the significant main effect of *group* found in time-frequency ROIs in the alpha band during task preparation and memory retrieval phases. Based on this congruence, we can conclude with high *confidence* (81) that the DAN model’s decision to classify subjects as patients or controls is based on the same aspects of the data that were revealed by the time-frequency analysis. Given that the detected abnormalities are oscillatory in nature and the DAN algorithm partially operates by convolution (82), it might have been specially suited to detect oscillatory signatures in the EEG. Because, similarly, decomposition of the EEG into the time-frequency domain is often accomplished by convolution of the EEG signal with complex Morlet wavelets, which was our method of choice for the time-frequency analysis in this study.

These results add to the rapidly growing body of literature reporting encouraging results in the use of machine learning to classify patients and controls in schizophrenia (46–52), with the ultimate goal of aiding and improving the challenging diagnosis of such heterogeneous disorder (83). Furthermore, the demonstrated interpretability of our model highlights that machine learning can be designed to serve not only as a diagnostic aid in classification, but also to probe the neurophysiological correlates of schizophrenia and, potentially, other psychiatric disorders.

### 4.4. Limitations and future directions

This study has some limitations. Our sample size was small, which may have affected statistical power. However, this is a consequence of the challenging goal of recruiting a homogeneous group of schizophrenia patients. Furthermore, our sample is constituted exclusively by males, which may limit the translational value of the study. Finally, although the accuracy of our DAN machine learning model is significant and provides additional information about the differences between patients and controls, it is not robust enough to support the direct diagnosis or classification of patients on its own.

Nevertheless, the machine learning and time-frequency results both suggest that in schizophrenia there is a significant impact on working memory processes during the task preparation and maintenance phases. Even in high performing patients that show no significant impact in behavioral performance or ERP correlates, when compared to healthy controls. Furthermore, the features studied could be combined with a broader set of features to support more accurate identification of patients. In that fashion, these techniques could be used as a diagnostic complement to more established clinical assessment methods to help in early detection or differential diagnosis of neuropsychiatric disorders with suspected oscillatory abnormalities. Moreover, the DAN model’s accuracy could still be further improved by enriching its input with relevant multimodal data. For instance, as we have argued that the oscillatory abnormalities in the alpha band may indicate an inhibitory and attention deficit, future research could design experiments that would not only include EEG, but also additional techniques such as pupillometry, to measure changes in attention and arousal (84), or non-invasive stimulation, to directly probe the role of inhibitory neural circuits during the task (85). Finally, based on the neurophysiological insight provided by our model, we further encourage the incorporation of interpretable models in schizophrenia research.

## Data availability statement

The original contributions presented in the study are publicly available. This data can be found here: https://gitlab.com/MaticKu/shizo.

## Ethics statement

The studies involving humans were approved by the Medical Ethics Committee of the Republic of Slovenia. The studies were conducted in accordance with the local legislation and institutional requirements. The participants provided their written informed consent to participate in this study.

## Author contributions

JB and GR: study conception and design. JB, RP-A, and IP: data collection. RP-A, MK, BŠkr, and IP: data analysis. RP-A, AO, BŠko, PP, KA-P, GR, and JB: results interpretation. RP-A, AO, and JB: manuscript writing. All authors contributed to the manuscript review and approved the submitted version.

## Footnotes

1
https://gitlab.com/MaticKu/shizo
