# Supplementary material for: Dense attention network identifies EEG abnormalities during working memory performance of patients with schizophrenia
Source: Front Psychiatry. 2023 Sep 25;14:1205119. doi: 10.3389/fpsyt.2023.1205119 (PMC10560761; doi:10.3389/fpsyt.2023.1205119)
Supplement: Supplementary file 1 [file Data_Sheet_1.docx]

Supplementary Material

**Dense Attention Network identifies EEG abnormalities during working memory performance of patients with schizophrenia**

**Ruben Perellón-Alfonso*, Aleš Oblak, Matija Kuclar, Blaž Škrlj, Indre Pileckyte, Borut Škodlar, Peter Pregelj, Kilian Abellaneda-Pérez, David Bartrés-Faz, Grega Repovš, Jurij Bon***

*** Correspondence:** Ruben Perellón-Alfonso: [ruben.perellon@ub.edu](mailto:ruben.perellon@ub.edu) and Jurij Bon: [jurij.bon@mf.uni-lj.si](mailto:jurij.bon@mf.uni-lj.si)

# Empirical evaluation of the machine learning models

## Simple feedforward networks

We began our empirical evaluation by implementing a series of simple feedforward neural networks, which took as input the vectors describing individual patients. At this point, we already performed initial experiments with Logistic Regression, where the baseline performance of 65% (*F_1_* score) was established. Feedforward (or dense) neural networks can be, when properly regularized, suitable for low-data scenarios. These neural networks are composed of layers, comprised of computational units – neurons. The whole neural network is trained via the process of backpropagation, an optimization procedure where errors obtained by comparing the predictions against the target (real) values are propagated and used to update the weights of the neural network.

## The attention mechanism

Recent advancements in natural language processing rely on the notion of neural attention, a simple mechanism which scatters the input signals and highlights only the parts of the feature space which are relevant to the task at hand. This concept is thoroughly explained elsewhere (Škrlj et al., 2020), therefore, we introduce here only the necessary ideas to understand this analysis. Originally, the attention mechanism was used for neural translation purposes, where a mapping between two sequences needed to be learned. Such, attention layer (La) can be formulated as:


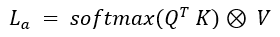


where Q, K and V are the query, key and value, respectively. The query and key sequence’s indices are thus associated with a given value. Here we explored how a similar idea performs on simple, feedforward neural networks and time-dependent inputs extracted from the reduced EEG data.

## Dense Attention Networks

The attention mechanism is inspired by the recently introduced language models (Devlin et al., 2019), and for our data it can be defined as:


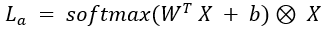


For the first layer, followed by standard dense layers with additional regularization in form of the dropout. The W thus corresponds to a weight matrix and X to the whole input time series. When used directly as output, such attention layers emit a probability distribution across the input space, offering a window into the inner workings of the neural network and a potential opportunity to identify whether some spurious correlations were learnt.

## Model interpretability

In terms of how models are interpretable, we can distinguish between two main interpretability types. Symbolic models, such as trees or similar, which yield conjuncts of features, are directly interpretable to a human observer. Statistical methods, such as neural networks are commonly interpreted post-hoc – their predictions are approximated one-by-one and aggregated. However, the attention mechanism offers an alternative, as it maintains a bijection with the input space throughout the training (the attention layer corresponds to the input space in a one-to-one manner). The main caveat is that if the network learns correlations which are not causal, the attention will similarly emphasize wrong parts of the input space. We explored qualitatively, whether the attention vectors, once aggregated across all correctly predicted classifications, highlight timepoints that are also relevant with respect to the experimental design. The attention mechanism, thus, offers one approach to understand which segments of the feature space are relevant. However, as the inputs are aggregated prior to being fed to a neural network, this mechanism does not help with explanations related to individual conditions. Here, we developed a different approach which tackles this issue in a different manner altogether. Note that six possible task conditions were measured. As these conditions represent potentially individual parts of the feature space, we can design an evaluation procedure, where the models are trained on subsets of the space of all conditions (results are summarized in supplementary table S3 and figure S1).

# Time-Frequency statistical analysis results of ROIs in the theta, beta and gamma frequency bands

A mixed design ANOVA with a within-subject factor condition (condition 2, condition 2+2, and condition 4), a within-subject factor task phase (preparation, encoding, maintenance, retrieval), and a between-subject factor group (patient vs. control) on average power in theta range revealed a significant main effect of task phase, F(3,84) = 50.25, p <.001, η^2^ = .067. A pairwise analysis with Bonferroni correction revealed that theta power was higher during encoding, M = 0.13, SD = 1.88, than during the maintenance, M = -1.13, SD = 1.81, p = .046. For beta frequency band, we found a significant main effect of task phase, F(3,84) = 3.53, p = .026, η^2^ = .018, as well as significant interaction between task phase and condition, F(6, 168) = 5.57, p < 001, η^2^ = .009. A pairwise analysis with Bonferroni correction revealed that beta power was higher during maintenance, M = -0.50, SD = 0.58, than during the retrieval, M = -0.69, SD = 0.68, p = .049. When task phase was held constant, beta power was different between conditions during the retrieval only, F(2, 56) = 9.10, p = .004, η^2^ = .044, but not during the other three task phases (p>0.05). A pairwise analysis with Bonferroni correction revealed that during the retrieval phase beta power was significantly more negative at condition 2+2, M = -0.88, SD = 0.65, when compared to condition 2, M = -0.64, SD = 0.66, p = .012, and condition 4, M = -0.55, SD = 0.71, p = .004. Finally, we found a significant main effect of task phase at gamma frequency band, F(3, 84) = 3.28, p = 025, η^2^ = .020. A pairwise analysis with Bonferroni correction revealed that gamma power was almost significantly different between maintenance and retrieval periods, p = .053.

# Supplementary References

Devlin, J., Chang, M. W., Lee, K., & Toutanova, K. (2019). BERT: Pre-training of deep bidirectional transformers for language understanding. NAACL HLT 2019 - 2019 Conference of the North American Chapter of the Association for Computational Linguistics: Human Language Technologies - Proceedings of the Conference, 1(Mlm), 4171–4186.

Škrlj, B., Džeroski, S., Lavrač, N., & Petković, M. (2020). Feature importance estimation with self-attention networks. ArXiv, 1.

**Table S1**. Demographics and clinical properties of study participants

| **Participant property** | **Patient group** | **Control group** |
| --- | --- | --- |
| Age [mean (SD)] | 28.1 (3.87) | 26.8 (5.54) |
| Years of education [mean (SD)] | 13.4 (1.12) | 14.4 (1.18) |
| Handedness [N] |  |  |
| Right | 14 | 13 |
| Left | 1 | 1 |
| Ambidextrous | 0 | 1 |
| Antipsychotic medication [N] |  |  |
| Aripiprazole | 4 | 0 |
| Olanzapine | 3 | 0 |
| Clozapine | 5 | 0 |
| Quetiapine | 2 | 0 |
| Risperidone | 3 | 0 |
| Paliperidone | 1 | 0 |
| Amisulpride | 2 | 0 |
| ICD-10 F code [N] |  |  |
| F 20.0 | 12 | 0 |
| F 25 | 3 | 0 |
| PSP [mean (SD)] | 53.73 (8.28) | N/A |
| EASE [mean (SD)] |  |  |
| Cognition | 35.5 (14.3) | N/A |
| Presence | 42.4 (13.9) | N/A |
| Body | 13.3 (15.3) | N/A |
| Transitivism | 18.7 (22.0) | N/A |
| Existential | 19.1 (14.8) | N/A |
| Hyperreflexivity | 2.1 (0.9) | N/A |
| PANSS [mean (SD)] | 77.07 (15.29) | N/A |

**Table S2**. Working memory task performance. H = hit rate; F = false alarm rate; K = memory capacity index.

| **WM performance** | **Experimental group** | **Control group** |
| --- | --- | --- |
| Condition 2 |  |  |
| Errors when different [mean (SD]) | 7.5 (4.2) | 11.4 (8.2) |
| Errors when same [mean(SD)] | 4.2 (4.2) | 5.8 (4.3) |
| All errors [mean(SD)] | 11.7 (6.9) | 17.2 (10.6) |
| Accuracy [mean(SD)] | 94.5 (3.3) | 91.7 (4.9) |
| H – F [mean(SD)] | 0.9 (0.07) | 0.84 (0.1) |
| K | 1.79 (0.13) | 1.67 (0.21) |
| Reaction time | 840.27 (403.45) | 764.10 (372.71) |
| Condition 4 |  |  |
| Errors when different [mean (SD]) | 27.8 (15.7) | 39.7 (22.3) |
| Errors when same [mean(SD)] | 12.9 (9.2) | 8.3 (5.7) |
| All errors [mean(SD)] | 40.7 (20.5) | 48.0 (23.2) |
| Accuracy [mean(SD)] | 81.0 (9.5) | 77.7 (10.9) |
| H – F [mean(SD)] | 0.64 (0.21) | 0.55 (0.22) |
| K | 2.47 (0.77) | 2.19 (0.86) |
| Reaction time | 990.21 (497.95) | 959.11 (472.68) |
| Condition 2+2 |  |  |
| Errors when different [mean (SD]) | 7.7 (7.1) | 14.8 (16.7) |
| Errors when same [mean(SD)] | 5.2 (3.7) | 7.9 (6.9) |
| All errors [mean(SD)] | 12.9 (9.8) | 22.7 (22.1) |
| Accuracy [mean(SD)] | 94.1 (3.6) | 89.4 (10.3) |
| H – F [mean(SD)] | 0.89 (0.09) | 0.79 (0.21) |
| K | 1.74 (0.19) | 1.59 (0.42) |
| Reaction time | 883.18 (435.30) | 776.83 (369.26) |
| Overall reaction time | 904.45 (451.60) | 833.24 (417.23) |

**Table S3**. Average performance metrics and standard deviation (in parenthesis) for each machine learning model tested in each subset of the feature space (i.e., experimental condition 2, 2+2 and 4 items; for both right and left visual hemifield presentation). CNN, convolutional neural network; DAN, dense attentional network; FFNN, feed forward neural network; KNN, K-nearest neighbour; LR, linear regression; RF, radio frequency machine learning; SVM, support vector machine; rbf, radial basis function.

| **Model** | **Condition** | **Accuracy** | **F1** | **Recall** | **Precision** |
| --- | --- | --- | --- | --- | --- |
| CNN | 2 left | 0.67 ± (0.03) | 0.7 ± (0.01) | 0.77 ± (0.11) | 0.65 ± (0.06) |
| CNN | 2 right | 0.71 ± (0.03) | 0.73 ± (0.02) | 0.77 ± (0.0) | 0.69 ± (0.03) |
| CNN | 2+2 left | 0.69 ± (0.11) | 0.72 ± (0.11) | 0.81 ± (0.16) | 0.65 ± (0.07) |
| CNN | 2+2 right | 0.71 ± (0.03) | 0.72 ± (0.03) | 0.73 ± (0.05) | 0.7 ± (0.02) |
| CNN | 4 left | 0.71 ± (0.14) | 0.73 ± (0.12) | 0.77 ± (0.11) | 0.69 ± (0.13) |
| CNN | 4 right | 0.71 ± (0.08) | 0.73 ± (0.12) | 0.81 ± (0.27) | 0.68 ± (0.01) |
| CNN | all | 0.69 ± (0.05) | 0.72 ± (0.05) | 0.81 ± (0.05) | 0.66 ± (0.04) |
| DAN | 2 left | 0.62 ± (0.05) | 0.62 ± (0.09) | 0.65 ± (0.16) | 0.6 ± (0.03) |
| DAN | 2 right | 0.75 ± (0.03) | 0.75 ± (0.05) | 0.77 ± (0.11) | 0.74 ± (0.01) |
| DAN | 2+2 left | 0.62 ± (0.05) | 0.66 ± (0.05) | 0.73 ± (0.05) | 0.59 ± (0.04) |
| DAN | 2 right | 0.63 ± (0.08) | 0.69 ± (0.06) | 0.81 ± (0.05) | 0.6 ± (0.06) |
| DAN | 4 left | 0.56 ± (0.14) | 0.65 ± (0.09) | 0.81 ± (0.05) | 0.54 ± (0.1) |
| DAN | 4 right | 0.75 ± (0.03) | 0.79 ± (0.02) | 0.92 ± (0.0) | 0.69 ± (0.03) |
| DAN | all | 0.69 ± (0.05) | 0.71 ± (0.07) | 0.77 ± (0.11) | 0.67 ± (0.03) |
| FFNN | 2 left | 0.77 ± (0.0) | 0.78 ± (0.01) | 0.81 ± (0.05) | 0.75 ± (0.03) |
| FFNN | 2 right | 0.79 ± (0.03) | 0.8 ± (0.02) | 0.85 ± (0.0) | 0.76 ± (0.04) |
| FFNN | 2+2 left | 0.67 ± (0.03) | 0.69 ± (0.03) | 0.73 ± (0.05) | 0.65 ± (0.02) |
| FFNN | 2+2 right | 0.67 ± (0.08) | 0.72 ± (0.05) | 0.85 ± (0.0) | 0.63 ± (0.08) |
| FFNN | 4 left | 0.67 ± (0.03) | 0.7 ± (0.05) | 0.77 ± (0.11) | 0.64 ± (0.0) |
| FFNN | 4 right | 0.67 ± (0.03) | 0.71 ± (0.0) | 0.81 ± (0.05) | 0.64 ± (0.04) |
| FFNN | all | 0.71 ± (0.03) | 0.72 ± (0.03) | 0.73 ± (0.05) | 0.7 ± (0.02) |
| KNN | 2 left | 0.62 ± (0.0) | 0.55 ± (0.0) | 0.46 ± (0.0) | 0.67 ± (0.0) |
| KNN | 2 right | 0.5 ± (0.0) | 0.55 ± (0.0) | 0.62 ± (0.0) | 0.5 ± (0.0) |
| KNN | 2+2 left | 0.54 ± (0.0) | 0.6 ± (0.0) | 0.69 ± (0.0) | 0.53 ± (0.0) |
| KNN | 2+2 right | 0.65 ± (0.0) | 0.71 ± (0.0) | 0.85 ± (0.0) | 0.61 ± (0.0) |
| KNN | 4 left | 0.54 ± (0.0) | 0.57 ± (0.0) | 0.62 ± (0.0) | 0.53 ± (0.0) |
| KNN | 4 right | 0.65 ± (0.0) | 0.67 ± (0.0) | 0.69 ± (0.0) | 0.64 ± (0.0) |
| KNN | all | 0.69 ± (0.0) | 0.71 ± (0.0) | 0.77 ± (0.0) | 0.67 ± (0.0) |
| LR | 2 left | 0.73 ± (0.0) | 0.72 ± (0.0) | 0.69 ± (0.0) | 0.75 ± (0.0) |
| LR | 2 right | 0.69 ± (0.0) | 0.71 ± (0.0) | 0.77 ± (0.0) | 0.67 ± (0.0) |
| LR | 2+2 left | 0.58 ± (0.0) | 0.56 ± (0.0) | 0.54 ± (0.0) | 0.58 ± (0.0) |
| LR | 2+2 right | 0.58 ± (0.0) | 0.65 ± (0.0) | 0.77 ± (0.0) | 0.56 ± (0.0) |
| LR | 4 left | 0.62 ± (0.0) | 0.67 ± (0.0) | 0.77 ± (0.0) | 0.59 ± (0.0) |
| LR | 4 right | 0.62 ± (0.0) | 0.64 ± (0.0) | 0.69 ± (0.0) | 0.6 ± (0.0) |
| LR | all | 0.65 ± (0.0) | 0.67 ± (0.0) | 0.69 ± (0.0) | 0.64 ± (0.0) |
| RF | 2 left | 0.6 ± (0.03) | 0.55 ± (0.01) | 0.5 ± (0.05) | 0.62 ± (0.06) |
| RF | 2 right | 0.62 ± (0.11) | 0.64 ± (0.1) | 0.69 ± (0.11) | 0.6 ± (0.09) |
| RF | 2+2 left | 0.44 ± (0.08) | 0.43 ± (0.0) | 0.42 ± (0.05) | 0.45 ± (0.07) |
| RF | 2+2 right | 0.58 ± (0.05) | 0.61 ± (0.05) | 0.65 ± (0.05) | 0.57 ± (0.05) |
| RF | 4 left | 0.38 ± (0.11) | 0.36 ± (0.15) | 0.35 ± (0.16) | 0.37 ± (0.13) |
| RF | 4 right | 0.58 ± (0.05) | 0.57 ± (0.1) | 0.58 ± (0.16) | 0.57 ± (0.04) |
| RF | all | 0.56 ± (0.08) | 0.58 ± (0.09) | 0.62 ± (0.11) | 0.55 ± (0.07) |
| SVM linear | 2 left | 0.73 ± (0.0) | 0.72 ± (0.0) | 0.69 ± (0.0) | 0.75 ± (0.0) |
| SVM linear | 2 right | 0.58 ± (0.0) | 0.59 ± (0.0) | 0.62 ± (0.0) | 0.57 ± (0.0) |
| SVM linear | 2+2 left | 0.54 ± (0.0) | 0.5 ± (0.0) | 0.46 ± (0.0) | 0.55 ± (0.0) |
| SVM linear | 2+2 right | 0.65 ± (0.0) | 0.67 ± (0.0) | 0.69 ± (0.0) | 0.64 ± (0.0) |
| SVM linear | 4 left | 0.62 ± (0.0) | 0.64 ± (0.0) | 0.69 ± (0.0) | 0.6 ± (0.0) |
| SVM linear | 4 right | 0.58 ± (0.0) | 0.56 ± (0.0) | 0.54 ± (0.0) | 0.58 ± (0.0) |
| SVM linear | all | 0.62 ± (0.0) | 0.62 ± (0.0) | 0.62 ± (0.0) | 0.62 ± (0.0) |
| SVM poly | 2 left | 0.54 ± (0.0) | 0.4 ± (0.0) | 0.31 ± (0.0) | 0.57 ± (0.0) |
| SVM poly | 2 right | 0.73 ± (0.0) | 0.72 ± (0.0) | 0.69 ± (0.0) | 0.75 ± (0.0) |
| SVM poly | 2+2 left | 0.62 ± (0.0) | 0.55 ± (0.0) | 0.46 ± (0.0) | 0.67 ± (0.0) |
| SVM poly | 2+2 right | 0.5 ± (0.0) | 0.48 ± (0.0) | 0.46 ± (0.0) | 0.5 ± (0.0) |
| SVM poly | 4 left | 0.5 ± (0.0) | 0.38 ± (0.0) | 0.31 ± (0.0) | 0.5 ± (0.0) |
| SVM poly | 4 right | 0.62 ± (0.0) | 0.62 ± (0.0) | 0.62 ± (0.0) | 0.62 ± (0.0) |
| SVM poly | all | 0.62 ± (0.0) | 0.58 ± (0.0) | 0.54 ± (0.0) | 0.64 ± (0.0) |
| SVM rbf | 2 left | 0.42 ± (0.0) | 0.44 ± (0.0) | 0.46 ± (0.0) | 0.43 ± (0.0) |
| SVM rbf | 2 right | 0.5 ± (0.0) | 0.48 ± (0.0) | 0.46 ± (0.0) | 0.5 ± (0.0) |
| SVM rbf | 2+2 left | 0.42 ± (0.0) | 0.4 ± (0.0) | 0.38 ± (0.0) | 0.42 ± (0.0) |
| SVM rbf | 2+2 right | 0.38 ± (0.0) | 0.38 ± (0.0) | 0.38 ± (0.0) | 0.38 ± (0.0) |
| SVM rbf | 4 left | 0.27 ± (0.0) | 0.24 ± (0.0) | 0.23 ± (0.0) | 0.25 ± (0.0) |
| SVM rbf | 4 right | 0.42 ± (0.0) | 0.44 ± (0.0) | 0.46 ± (0.0) | 0.43 ± (0.0) |
| SVM rbf | all | 0.58 ± (0.0) | 0.62 ± (0.0) | 0.69 ± (0.0) | 0.56 ± (0.0) |

**
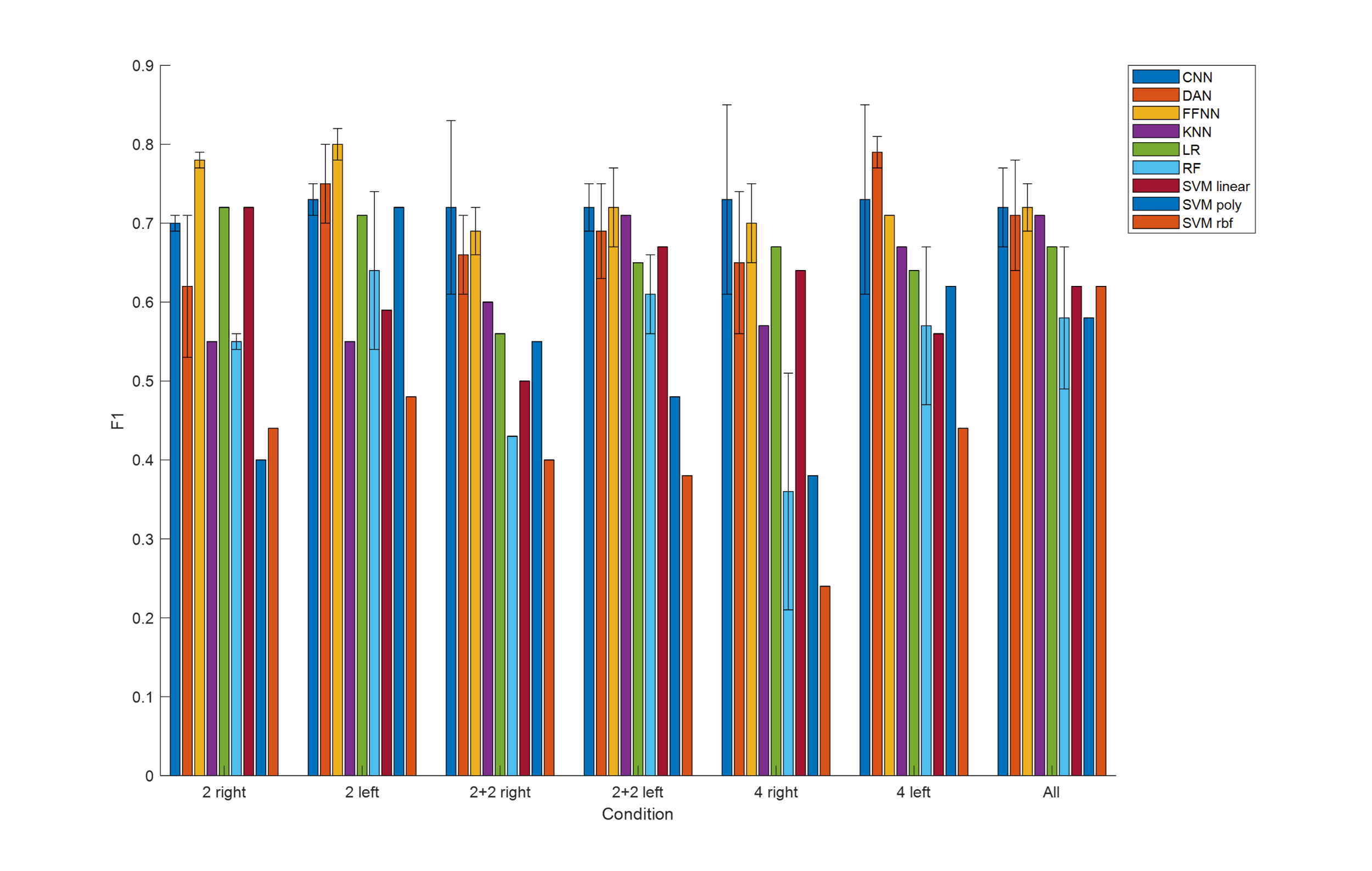
**

**Figure S1**. Bar graph ilustrating average performance metrics (black bars show standard deviations) for each machine learning model tested in each subset of the feature space (i.e., experimental condition 2, 2+2 and 4 items; for both right and left visual hemifield presentation). CNN, convolutional neural network; DAN, dense attentional network; FFNN, feed forward neural network; KNN, K-nearest neighbour; LR, linear regression; RF, radio frequency machine learning; SVM, support vector machine; rbf, radial basis function.


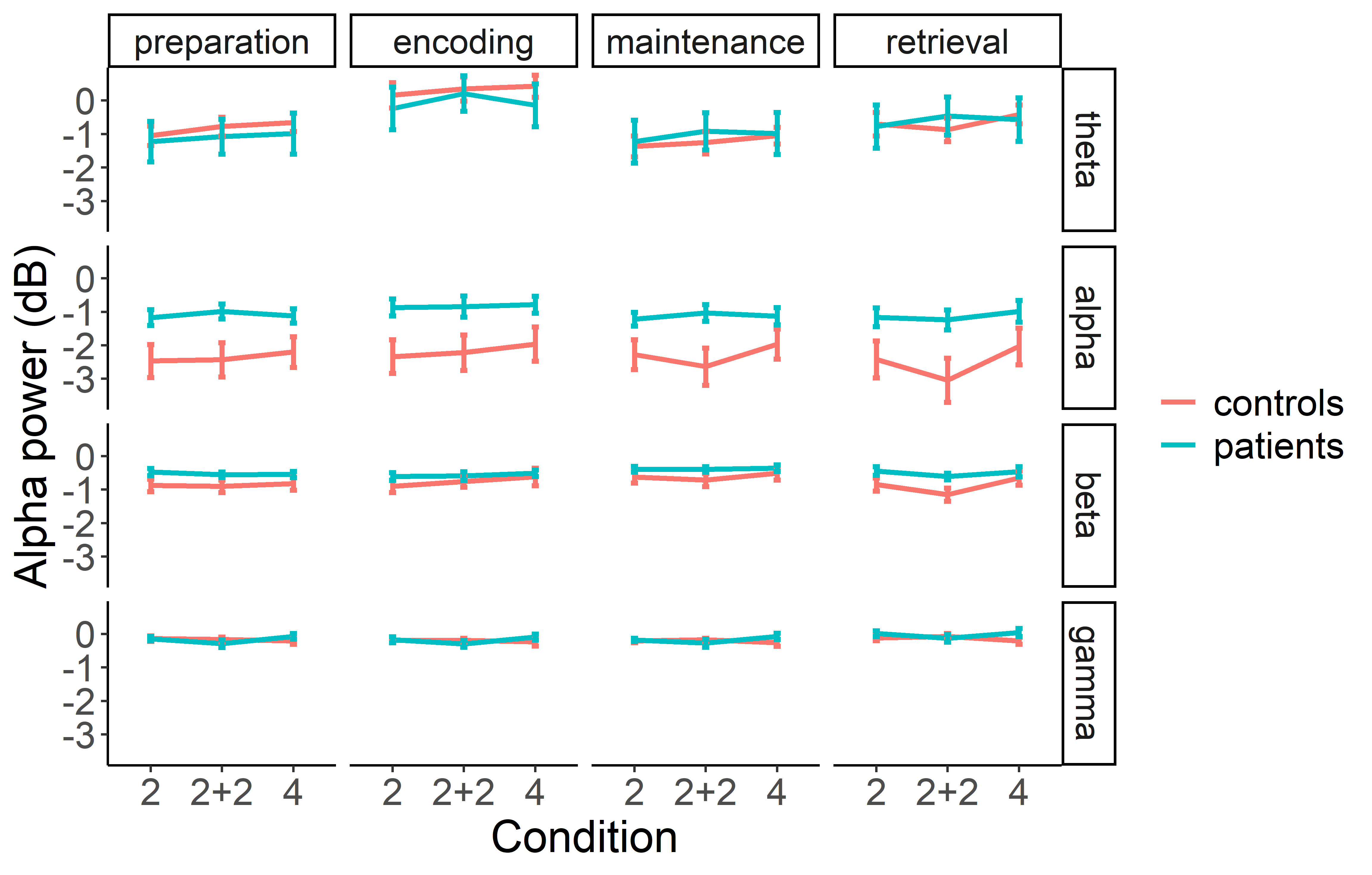


Figure S2. Average power values for patients (blue line) and controls (red line) in four WM task phases (preparation, encoding, maintenance, and retrieval) and four frequency bands (theta, alpha, beta, and gamma). The error bars represent standard error of mean.
